# Effects of environmental metal and metalloid pollutants on plants and human health: exploring nano-remediation approach

**DOI:** 10.1007/s44154-024-00156-y

**Published:** 2024-05-23

**Authors:** Priyadarshani Rajput, Abhishek Singh, Shreni Agrawal, Karen Ghazaryan, Vishnu D. Rajput, Hasmik Movsesyan, Saglara Mandzhieva, Tatiana Minkina, Athanasios Alexiou

**Affiliations:** 1 Academy of Biology and Biotechnology, Southern Federal University, Rostov-On-Don, Russia; 2 Faculty of Biology, Yerevan State University, 0025 Yerevan, Armenia; 3 Department of Biotechnology, Parul Institute of Applied Science, Parul University, Vadodara, Gujarat India; 4 Department of Science and Engineering, Novel Global Community Educational Foundation, Hebersham, NSW 2770 Australia; 5 AFNP Med, 1030 Vienna, Austria

**Keywords:** Human health, Metal, Nanoparticles, Remediation, Toxicity

## Abstract

Metal and metalloid pollutants severely threatens environmental ecosystems and human health, necessitating effective remediation strategies. Nanoparticle (NPs)-based approaches have gained significant attention as promising solutions for efficient removing heavy metals from various environmental matrices. The present review is focused on green synthesized NPs-mediated remediation such as the implementation of iron, carbon-based nanomaterials, metal oxides, and bio-based NPs. The review also explores the mechanisms of NPs interactions with heavy metals, including adsorption, precipitation, and redox reactions. Critical factors influencing the remediation efficiency, such as NPs size, surface charge, and composition, are systematically examined. Furthermore, the environmental fate, transport, and potential risks associated with the application of NPs are critically evaluated. The review also highlights various sources of metal and metalloid pollutants and their impact on human health and translocation in plant tissues. Prospects and challenges in translating NPs-based remediation from laboratory research to real-world applications are proposed. The current work will be helpful to direct future research endeavors and promote the sustainable implementation of metal and metalloid elimination.

## Introduction

The long-term persistence and toxicity of metal and metalloid (HMMs) pollutants make soil pollution a major concern (Uchimiya et al. 2020). Agrochemicals, potentially harmful metals, and an overabundance of nutrients are being added to the soil as a result of the fast expansion of human and technogenic activities (Midhat et al. 2019). HMs are present in agricultural soils because of extensive use of chemical fertilizers, farmyard manure, sewage sludge, atmospheric deposition, and the rapid increase of industrialization (Rastegari Mehr et al. 2021). Over 20 million hectares (ha) of land worldwide is polluted with cadmium, arsenic, mercury, nickel, zinc, lead, copper, and chromium (Liu et al. 2018). The ATSDR (Agency for Toxic Substances and Disease Registry) states that four heavy metals—cadmium, arsenic, lead, and mercury—are extremely harmful to both humans and plants (Mansoor et al. 2021). HMMs s have a potential to enter plant systems and contaminate food chains (Yang et al. 2021; Zheng et al. 2021). This fact presents a severe risk to both food quality and human health.

Soil is essential for agricultural development because it allows plants to absorb nutrients (Bongaarts 2009). Although farming is vital to human survival and improves our quality of life, it frequently has negative effects on the environment (Porter and Sachs 2020). A highly debated issue in the scientific literature, the ever-increasing demand for natural resources by an ever-increasing human population poses a dilemma between the advantages of arable land and the need for sustainable land management (Cocklin et al. 2007; Zulfiqar et al. 2021). Sustainable development, people's livelihoods, and quality of life are all endangered by land contamination (Martin et al. 2016; Zulfiqar et al. 2019b; Haider et al. 2022). With an increasing population and less arable land because of technological activity, soil preservation is of the highest priority. Air, water, and soil pollution are major problems all over the world that endanger people and ecosystems (Madhav et al. 2020). Many studies have documented the harmful impacts of HMMs on many species, including plants, human cells, and microbes (Alengebawy et al. 2021a). According to Ali and Rawlins (2021) and J. Zhou et al. (2021b), HMMs have phytotoxic effects that damage plant cells' structure and growth. Cellular oxidative stress, caused by denaturation of proteins and DNA damage, causes cells to die when exposed directly to HMMs (Chen et al. 2021).

Various *in-situ* and *ex-situ* remediation methods have been employed to reduce HMMs content in polluted soils in recent decades. These include bioremediation, surface capping, vitrification, electrokinetic extraction, solidification, soil flushing, and electrokinetic extraction (Liu et al. 2018; Kumar et al. 2019; Hu et al. 2021). Conventional physicochemical remediation methods include discharging toxic chemicals into the environment untreated over time (Kumar et al. 2019). In addition, bioremediation utilizes biological processes, including plants and microbes, to reduce the harmful effects of HMMs-related toxicity in polluted areas. Nevertheless, there are numerous drawbacks to these methods. For example, they are expensive to operate, take a long time to complete the process, do not provide an ideal environment for microbes, increase the bioavailability of contaminants on both spatial and temporal scales, and are performed poorly in real-world field settings (Singh et al. 2013; Hou et al. 2020; Rahman and Singh 2020). The efficacy, operating cost, time needed, geography of the contaminated site, characteristics of the soil pollutant, public acceptability, and readiness for execution of these conventional remediation methods determine their applicability. To immobilize HMMs on a wide scale, new recyclable, nontoxic, inexpensive, and environmentally friendly adsorbents need to be developed (Alengebawy et al. 2021a). In response to these difficulties, a new and potentially useful approach called nanoremediation has evolved, which involves cleaning up polluted environments with designed nanoparticles (NPs) (Ganie et al. 2021).

Plants and the microbiome they inhabit are both negatively affected by HM in the soil. Their stress causes reactive oxygen species (ROS) to be formed, which harm the organism's essential macromolecules and impact the ecology and nutrition of plants and crops (Samanta et al. 2024). When Cu-contaminated soil is mixed with natural soil, for instance, it can interfere with plants natural growth processes by negatively impacting their biochemical reactions and physiological processes (Saleem et al. 2020). Plant species, heavy metal type, and physiochemical characteristics all play a role in how HM harms plants (Chaplygin et al. 2021). Soil HMMs and HMMs contamination may potentially affect plant and microbiome diversity, abundance, and genetic composition, according to certain research (Rajput et al. 2022a, b). There is an increase in oxidative stress due to ROS and a decrease in enzymatic activities, biomass, and agricultural productivity (Padmavathiamma and Li 2007; Dalcorso et al. 2010).

Site stabilization can help minimize the mobility, availability, and leaching of HMMs at remote locations, preventing their spread (Shanker et al. 2005; Kopittke and Menzies 2006; Sampaio et al. 2016; Jinadasa et al. 2016).Thermal treatment, pump-and-treat, chemical oxidation, and newer technologies like nanoremediation are a few examples of the methods used to restore polluted areas (Lim et al. 2014; Mukhopadhyay et al. 2022b; Boregowda et al. 2022). Nanoremediation is a more efficient and cost-effective way to clean up contaminated areas. A high surface area-to-mass ratio, unique electrical and catalytic capabilities, and responsiveness are all characteristics of NPs (Corsi et al. 2018). Environmental remediation is aided by NPs, mainly through catalysis and chemical reduction. In addition, NPs have been used in removal methods based on adsorption because of their large surface area, evenly distributed active sites, and adaptable coating changes (Corsi et al. 2018; Guerra et al. 2018). NPs are ideal for water and soil remediation because of their small particle size and dispersion capability (Marcon et al. 2021; Mariyam et al. 2024). In water nanofiltration the membranes based on NPs are also used, which successfully block the passage of bigger particles through the pores of the membrane (Singh Sekhon 2014; Zhu et al. 2017, 2021; Su et al. 2019). In addition, the interaction with the membrane makes it easier to separate smaller molecules. Nanomaterials used for water, soil, and air remediation include metal oxides, carbon nanotubes, quantum dots, and biopolymers (Rastogi et al. 2017). Main aim of this review to highlight current research and approaches used in environmental nanotechnology and to analyze the uses of different NPs in HMMs with ther detoxification. After that, discuss the many ways to interaction of NPs with plants and soil biota at physiobiochemical and molecular level to mitigated the cocnetation and toxicity effects of HMMs that further also affected the human healths.

## Global status of HMMs problem

Both naturally occurring and chemically produced HMMs can find their way into ecosystems. Precipitation, snowfall, volcanic eruptions, and wildfires are examples of natural sources; fertilizers, fuel combustion, mining, construction, deforestation, and many industrial operations are examples of man-made sources of HMMs. (Licata et al. 2004). Humans are surrounded by common elements, one of which is arsenic. It is the twelfth most common element in the human body, the twentieth most common element in the Earth's crust, and the fourteenth most common element in the water itself (Jomova et al. 2011).

According to the US Environmental Protection Agency (EPA), Australia has a concentration of arsenic (As) above 10,000 mg per kilogram hacters, and 41% of the world's 1.4 million sites poisoned with arsenic are in the US (Smith et al. 2009). In addition to exceeding the US EPA’s recommended limits of 10 parts per billion for arsenic concentration, several shallow reservoirs and bore wells in Pakistan are arsenic-polluted (Malik et al. 2009). Arsenic contamination of groundwater has been found in several countries around the globe (Bhattacharya et al. 2007). There is cause for worry over the toxicity of groundwater in South Asian countries. This phenomenon has been noted in several regions, including the Red River Delta in Vietnam, the Mekong Basin in Cambodia and Vietnam, the Terai Belt in Nepal, and the Bengal Basin in India, Bangladesh, and Pakistan. The outcome is that at least 100 million individuals in these nations are at risk of getting cancer and other disorders linked to arsenic. Disposal of pentavalent arsenic is less of a hassle compared to trivalent arsenic. An enormous increase from the 1950s, global Cr production has reportedly reached 105.4 million metric tons since the start of the industrial revolution (Bissen and Frimmel 2003). Because of its low absorption and translocation rates by plants, Cr has largely gone unrecognized in pollution studies. As a result, phytotoxicity and the accumulation of Cr in food chains are rare occurrences in natural environments.

As a HMMs with devastating impacts on all forms of life, cadmium is a major environmental concern. At pH 4.5 and 5.5, it is more mobile than zinc. However, it becomes stationary in the pH range beyond 7.5. Soil Cd concentrations in the UK have been steadily increasing over the last 130 years, with the most dramatic rise occurring in the last 20 years (Tchounwou et al. 2012). According to a data source, environmental exposure to cadmium has been especially problematic in Japan due to the large consumption of rice grown in irrigation water contaminated with cadmium (Martin et al. 2007).. Lead is a harmful environmental contaminant that has a devastating effect on many bodily systems. Much of the housing stock built before 1978 contains paint that contains lead. In 1978, lead-based paint was criminalized in the United States by the federal government. Lead usage was further limited by the 2003 EU Restriction of Hazardous Substances Directive (Martin et al. 2007).

## Sources of HMMs

Scientists separated the sources of HMMs into two primary types: natural and synthetic. Anthropogenic sources include industry, agriculture, mining, and residential effluents, whereas natural sources include sedimentary rocks, volcanic eruptions, soil formation, and rock weathering (Sutkowska et al. 2020). However, soil anthropogenic and geogenic contamination can be effectively characterizedusing pollution indicators. However, it should be mentioned that despite the application of advanced research techniques, source apportionment may be challenging in many circumstances (Alloway 2013). Sedimentation of aerosol particles, rainfall with HMMs, and agrochemicals are only some of the HMMs sources and origin variations mentioned by Alloway (Hou et al. 2020). HMMs like Cd, Pb, Cu, and Zn were the primary focus of the study, even though numerous other metals were also mentioned.

### HMMs natural sources of HMMs

Igneous rocks and sedimentary rocks are generally believed to be the most prevalent natural sources of heavy metals. The ranges of heavy metal concentrations, measured in parts per million (ppm), are found in igneous and sedimentary rocks. It has been discovered that various rock types contain varied quantities of the same elements and that these proportions also change from one rock type to the next (Bradl 2005). This is true even for the components that were detected in the same rock type. The type of rocks and the circumstances of the surrounding ecosystem are two factors that can impact the concentration of heavy metals (Shahid et al. 2014). In addition, the formation of soil is thought to be one of the primary reasons for the accumulation of heavy metals, along with the deposition of sediments in rivers.

### Anthropogenic HMMs sources of HMMs

Heavy metals can be found in wastewater that comes from anthropogenic sources such as industries, agriculture, and mining. These sources significantly contribute to the increase in the concentration of heavy metals and pollution in the ecosystem, for example, smelting, which leads to the release of Cu, Zn, and As; insecticides, which contribute to the release of As; the burning of fossil fuels, which produces Hg; and car exhaust, which assists in the release of Pb (Shahid et al. 2014; Saleh and Aglan 2018). In addition, regular human activities like farming, industrial operations, and manufacturing disrupt the natural equilibrium of the biosphere (He et al. 2005).

### Agricultural HMMsSources of HMMs

**Fig. 1:**
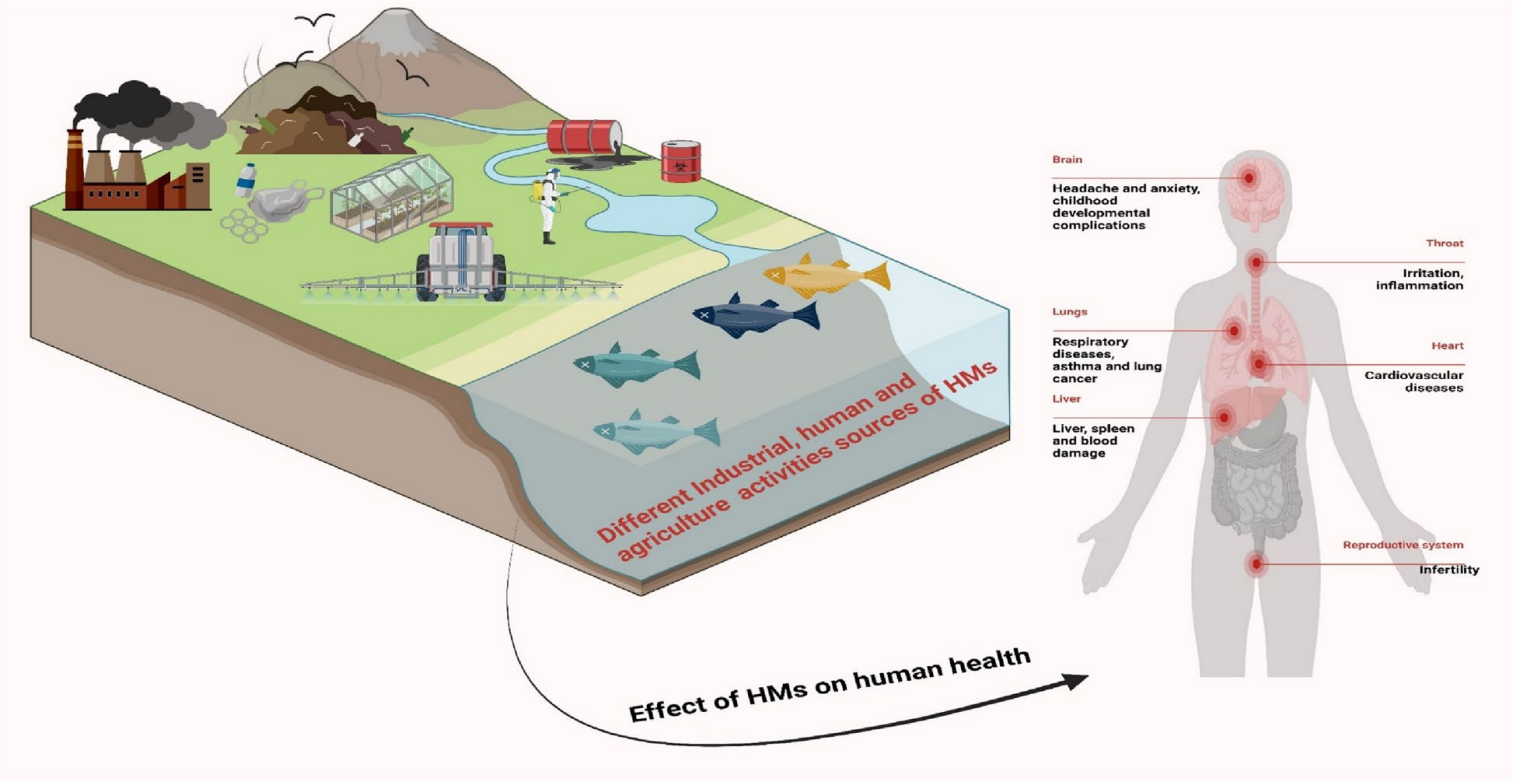
Different sources of HMMs and their accumulation in the environment with negative effect on human health

HMMs have been a part of Earth's crust ever since the planet was formed. Furthermore, as can be seen in Fig. 1, the most significant natural causes responsible for HMMs pollution in agricultural soils include soil erosion, floods, volcanic activity, sediment resuspension, metal corrosion, geological weathering, and metal evaporation from soil and wastewater. Due to rapid industrialization and urbanization, heavy metal levels have risen dramatically in recent years. The pollution by HMMs in terrestrial settings, especially agricultural fields, has become a worrisome concern for the developing world (Uchimiya et al. 2020). Anthropogenic activities, e.g., foundries, mining, smelting, leather industries, sewage, leaded paints, combustion of crop residues, spillage of petroleum distillates, automobiles, landfills, animal manure, wastewater irrigation, and runoff from different factories, are primary reasons for HM pollution (Yan et al. 2018; Li et al. 2019). Pesticides, fertilizers, herbicides, and other crop enhancement products are secondary sources of HMMs in agricultural fields (He et al. 2019; Liu et al. 2020a). Since the HMMs are not biodegradable, they will remain in the soil indefinitely without being broken down by the normal physiochemical processes. HMMs can enter the food chain when plants absorb them together with other nutrients and water from the soil (Ali and Khan 2019). Soil quality is negatively impacted and agricultural land is rendered unusable because HMMs enhance the impact of soil emissions by creating chelates with organic pollutants and modify the soil features, viz. pH, porosity, natural chemistry, and color (Uchimiya et al. 2020).

Fertilizers boost plant development and yield by adding organic matter to the soil and supplying it with the various nutrients plants need to thrive. Therefore, fertilizers increase soil productivity (Alengebawy et al. 2021b). There are two main types of fertilizers: organic (natural) and inorganic (man-made). After the anaerobic digestion (AD) process, ammonium fertilizers (sulfate and nitrate) are created as organic or biofertilizers (Cai et al. 2019). Chemically made or synthetic fertilizers, often known as inorganic fertilizers are a combination of inorganic components and chemical compounds (Chen et al. 2020). HMMs in the soil are a direct result of the use of fertilizers, both organic and artificial. Phosphorus is a key ingredient in many types of fertilizers, but it also contributes significantly to heavy metal buildup when applied to soil (Bolan et al. 2003). Phosphate rocks, which are formed when water-insoluble phosphorus fertilizers are used, are crucial in the immobilization of metals in soil through the precipitation of metal phosphates (Ai et al. 2020). Heavy metal deposition in agricultural soils from excessive fertilizer usage affects soil fertility, stunting plant development and crop yields (Wang et al. 2020a, b, c). It is highly difficult to repair the soil ecosystem once HMMs pollute the soil. Long-term usage of fertilizers increases the accumulation potential of Cu, Zn, and Cd in farmland soil (Fan et al. 2018). The primary forms of inorganic fertilizers that contribute to the release of heavy metals in agricultural soil and are then taken up by plants include phosphate fertilizers, liming materials, and bio-fertilizers (Liu et al. 2020b). As a result, they are consumed by animals and eventually by people (He et al. 2005).

## Impact of HMMs on plant and human health

### Impact of HMMs on plant health

Plant growth and development are stunted by HMMs since their density is five times that of water (Nagajyoti et al. 2010). Although HMMs are ubiquitous in plant-soil interactions, their concentration varies across geographic locations (Khlifi and Hamza-Chaffai 2010). Although HMMs are needed at low concentrations for the proper operation of many plant organs, they become hazardous to plants when their levels rise above the safe threshold. As time goes by, the HMMs reputation as important pollutants only grows (Rahman Md Ashfaqur Rahman Md Asiful Islam Shah Alam Zahidur Rahman et al. 2019). While some metals, such as Cu, Mn, Fe, Zn, Co, and Ni, are essential for plant growth as micronutrients, others, such as Cd, Cr, and Pb, are of little use to plants and can be toxic if present in excess for an extended period (Fig. 1) (Vardhan et al. 2019). When plants drink from adventitious roots, the HMMs can penetrate the plant, where they can stunt development, disrupt vital biological processes like photosynthesis, and ultimately harm the plant's health (Ali et al. 2014). A recent study reported that Al poisoning may lead to radial cell proliferation and growth retardation of roots in barley (Zelinová et al. 2011).

The accumulation of ROS, such as hydrogen peroxide, is responsible for these morphological alterations by inducing cellulose deposition and cell death. The toxicity of Al can cause damage to the plasma membrane, a delay in cell division, the generation of ROS, a shift in calcium homeostasis, cytoskeleton damage, and a reduction in plant development (Shetty et al. 2021). Similarly, black gram *Vigna mungo* (L.) Hepper showed signs of oxidative damage, cellular damage, and root injury because of Al toxicity (Chowra et al. 2017). Another study by Piršelová et al. (2016) found that the absorption of Cd lowers photosynthesis in plants due to a lack of ferric ions and the replacement of magnesium ions in chlorophyll. High amounts of Cd in soil are poisonous to plants, and their detrimental effects on growth may be observed on both the physiological and morphological levels. Cd toxicity is characterized by a decrease in mitotic activity in meristematic cells, which might slow plant development (Fuertes et al. 2016). Due to their toxicity, HMMs significantly interfere with plant uptake of vital nutrients from the soil. For example, an excess of Cr in soil can reduce plant uptake of calcium, phosphorus, magnesium, and iron by forming insoluble complexes and hiding the absorption site (Charles and Onyema 2016). Cr can affect the nutritional balance in plants, although in *Citrullus* plants, transporting Cr to different sections resulted in increased concentrations of phosphorus and manganese and decreased concentrations of iron, zinc, copper, and sulphur in leaves (Sharma et al. 2020).

### Impact of HMMs on human health

Human exposure to As, Pb, Cr, and Cd is the most prevalent kind of toxicity associated with HMMs. Ingestion of Pb-based paints, air or water pollution, and poorly coated food containers are all potential causes of HMMs poisoning (Ahmed Khoso et al. 2021). Exposure to HMMs can induce multiple serious human diseases, viz., cancer, Parkinson, Alzheimer, renal pathology, respiratory issues, children’s mental disorders, vision problems, depression, dementia, and neurological disorders (Fig. 1) (Satarug et al. 2010). HMMs are extremely persistent inside the human body following ingestion of contaminated foods (Chandra et al. 2001). Kumar et al. (2019) found that chronic exposure to Cd had negative effects on male reproductive health, including hormone synthesis, sperm motility, semen quality, and spermatogenesis. Multiple research projects have found that exposure to Cd has harmful effects on women's reproductive health (de Angelis et al. 2017; Massányi et al. 2020).

## Uptake and translocation of HMMs in plants from the soil

Increasing global growth and urbanization have increased the risk posed by HMMs to ecological systems (Zhou et al. 2021c; Rajput et al. 2021; Singh et al. 2023a). A rise in the concentration of HMMs in landscapes is detrimental to plant growth, agricultural productivity, and the well-being of humans and animals (Rolka et al. 2020). Important HMMs include Zn, Cu, Fe, and Mn, while irrelevant HMMs include Pb, As, and Se. Although crops need these elements for growth and development, their elevated levels in the soil lower agricultural yields (Żołnowski et al. 2021). Irrelevant HMMs like Cd, Hg, and Pb are not used by the farming industry for a specific biological function (Feng et al. 2021). The detrimental impacts of HMMs on plant development result in chlorosis and necrosis, which ultimately lead the plants to perish due to an insufficient level of chlorophyll and the closing of stomata (Rono et al. 2022). Furthermore, being exposed to polluted soil, water, and agricultural spray can cause an accumulation of such pollutants in crops, which can lead to health issues in humans such as cancer and kidney disease (Khan et al. 2020; Faizan et al. 2023).

**Fig. 2:**
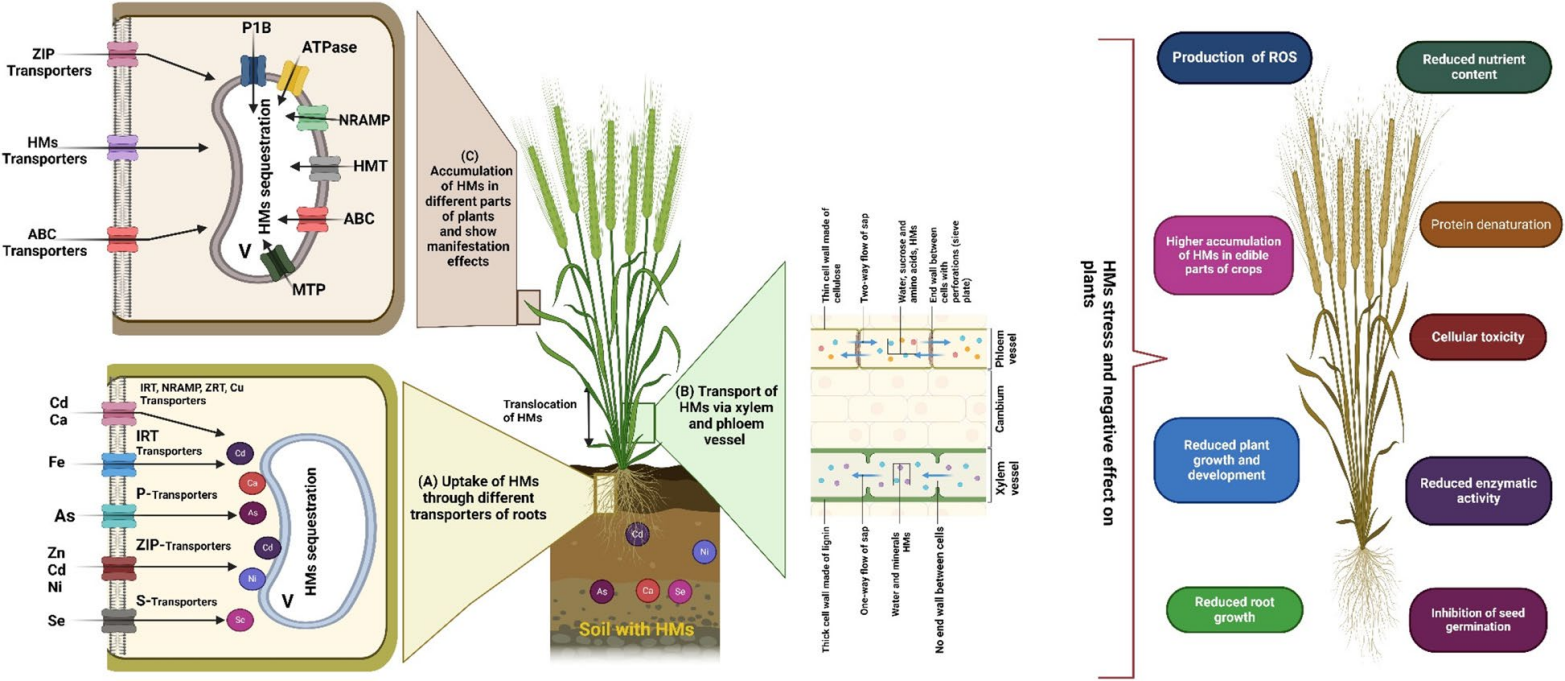
Diagrammatic reseparation of (**A**) uptake of HMMs by plant roots from soil and (**B**) translocation via xylem and phloem vessel through (**C**) different cellular transporters in plant cells. The accumulation also causes various negative effects on plant’s health

Plants use protein transporters in their membranes to absorb both essential and non-essential HMMs. Ca and Cd are carried into plants by a variety of transporters, including aquaporin, IRT, NRAMP, Cu-transporter, ZRT, and low-affinity divalent ions (Fig. 2) (Williams et al. 2000; Zhou et al. 2021a). Transporters of HMMs, such as P1B-type ATPase, HMT, NRAMP, and cation diffusion transporters, are found in vacuoles, Golgi bodies, and the endoplasmic reticulum when HMMs invade plant cells (Williams et al. 2000; Zhou et al. 2021a). The processes that eliminate or lessen the negative effects of HMMs include phytochelatins (PCs), metallothioneins (MTs), HM isoprenylated plant proteins (HIPPs), chemical substances, ligands, and compartmentalization (Fig. 2) (Khan et al. 2020; Feng et al. 2021; Rono et al. 2022).

### Arsenic (As)

The levels of the deadly metalloid arsenic (As) in soil are rising as a result of a combination of naturally occurring and human-induced processes, including mining (Fitz and Wenzel 2002). On average, there is 1.8 mg/kg of arsenic in the Earth's crust, presenting it as an almost minor component. Arsenopyrite (FeSAs), enargite (Cu_3_AsS_4_), orpiment (As_2_S_3_), realgar (As_2_S_2_), and others are among the most common minerals that contain arsenic (Masue et al. 2007; Zhao et al. 2021). Anaerobic (low-oxygen) groundwater settings often include arsenite As(III), a reduced form of arsenic that is more soluble and mobile in water (Masue et al. 2007). The oxidised form of arsenic is known as arsenate, As(V), or H_2_AsO_4_ − or HAsO_4_^2−^, depending on the pH (Yadav et al. 2021). The As(III) state is especially problematic because it is more hazardous and has a tendency to remain undissociated (e.g., H_3_AsO_3_) in groundwater throughout a wide pH range (Sarkar and Paul 2020). As is present in a variety of oxidation stages and at varying concentrations in soil (Panda et al. 2010). Both inorganic and organic substances of As in the environment are detrimental to plant development, while As(III), an inorganic form, is more hazardous and soluble. As in groundwater systems migrates and transforms in large part due to metal-reducing bacteria. Páez-Espino et al. (2009) discovered that arsenic-reducing bacteria had two distinct routes for arsenic reduction. Among these processes, one involves the ArrA proteins and anaerobic respiration, where arsenate serves as a terminal electron acceptor (Huber et al. 2000; Malasarn et al. 2004). Therefore, arsenate is reduced. Another is arsenic detoxication, which occurs when the cell uses its ArsC proteins to convert As(V) to As(III) (Xu et al. 1998; Achour et al. 2007). According to Aromokeye et al. (2021) and Dalla Vecchia et al. (2014) iron-reducing bacteria utilise soluble iron(III) complexes and iron(III) hydrogen oxides as the final electron acceptors in anaerobic respiration. In systems with high arsenic concentrations in aquifers, iron (hydrogen oxide) is the primary arsenate carrier (Xie et al. 2009). An essential key to manage arsenic enrichment in groundwater is the reduction and dissolution of iron (hydrogen) oxides, which is followed by the reduction of As(V) to more mobile As(III) (Xie et al. 2008). Microbes may be able to transport electrons to insoluble Fe(III) oxides outside of cells with the use of either endogenous or external electron shuttles (Taillefert et al. 2007; Von Canstein et al. 2008). Microbes decrease electron shuttles before they diffuse out of the cell. Giving Fe(III) oxide electrons causes it to revert to its oxidised condition, completing the cycle. Plants absorb As predominantly in an inorganic state (As(III) and As(V)) via transporters since it depends on accessibility (Panda et al. 2010). The plant experiences a variety of physiological, structural, and optical changes during uptake. Since several essential element transporters affect plant absorption, it is difficult to control their uptake (Saleem et al. 2022). Long-term exposure alters metabolic rate, phenotype (growth reduction), and physiology (chlorosis) (Neidhardt et al. 2015; Kofroňová et al. 2020). It may have negative impacts on plant growth that can lead to plant death, including the generation of ROS, protein breakdown, chlorosis, tissue necrosis, and the loss of vital intake of nutrients (Solórzano et al. 2020; Fitz and Wenzel 2002; Alengebawy et al. 2021a). The structural similarity between plant and element transporters allows plants to absorb the poisonous metalloid As and move it to various locations inside the plant. Plants take up As (III), which is similar to silicic acid, through aquaporins, while As (V), which is similar to phosphate which enters plants by phosphate transporters (Thounaojam et al. 2021). Toxic effects on plant development and yield are caused by various forms of arsenate that are translocated to the shoot through xylem after absorption; however, the effect is dependent on the effective concentration of free arsenate anion at a specific cellular organelle or location. Thus, transporters play a pivotal function as the primary regulator of As transportation in different plant tissues and cell compartments (Abbas et al. 2018a). To prevent As buildup and stress in plants, it is crucial to comprehend the specific functions of the many transporters engaged in As absorption and transit. The transporters covered include phosphate transporters, nodulin 26-like intrinsic proteins, plasma membrane intrinsic proteins, tonoplast intrinsic proteins, C-type ATP binding cassette transporters, arsenical resistance 3 transporter, inositol transporters, multidrug and toxic compound extrusion transporters, and natural resistance-associated macrophage protein transporters (Thounaojam et al. 2021). The root cells of plants absorb arsenate and arsenite by different mechanisms: phosphate transporters and NIP proteins, respectively. Arsenate is converted to arsenite by the arsenate reductase HAC1 (Zhang et al. 2021). Two more routes are involved in the detoxification of arsenite. First, it can be extruded via unknown transporters, most likely aquaporins. Another way is that ABCC proteins entrap arsenite in the vacuole as PCs-As(III) (Zhang et al. 2021).

### Nickel (Ni)

Plants require the mineral nickel (Ni), but just in small amounts (0.05 to 10 mg kg^−1^ dry weight), as high levels of Ni in the ecosystem are harmful to the development and growth of plants (Ahmad et al. 2011). It has been demonstrated that elevated Concentrations of Ni in crops cause growth inhibition, encourage the senescence of leaves, decrease nitrogen absorption, and disturb the concentration of Fe (Bhalerao et al.). While the amount of Ni in soil differs based on the circumstances, it is often greater than 20 mg kg^−1^ (Khanlari and Jalali 2008). Contaminated soils have 20–30 times higher Ni^2+^ levels than clean soils. Ni^2+^ levels rise as a result of human endeavors like phosphate fertilization, pesticide usage, burning of coal and oil, industrial emissions, and mineral exploitation (Kananke et al. 2014). Ni may be actively diffused or transferred within the vegetative system by the roots (Rue et al. 2020). After being transferred into the xylem, Ni moves from the root system towards the leaves and shoots (Chen et al. 2017). Ni is a contaminant that plants both actively and passively absorb and transport via xylem and due to its detrimental effects, Ni directly damage DNA, protein deterioration, and suppression of enzyme function.

### Chromium (Cr)

Cr is present in both the seas and the earth's outermost layers. The transition metal Cr is commonly found in the hexavalent Cr(VI) and trivalent Cr(III) oxidation states. Cr(VI) has a less volatile nature than Cr(III), making it more transportable but also more harmful (Dalal and Reddy 2019). The physiology of plants absorbing Cr from the soil may be adversely affected (Panda et al. 2010).

Studies on the specific pathways of Cr absorption and translocation have not been validated. Yet substances combined with organic acids enable roots to absorb them (Hayat et al. 2012). When crops take Cr through their roots, only a small amount of it enters different tissues and organs (Oliveira 2012). Raising of *Pisum sativum* L. in a solution containing potassium dichromate increased the concentrations of Cr in numerous plant organs (Tiwari et al. 2009). Cr exposure can affect a plant's overall moisture content and physiological activity from the time of germination until the roots, stems, and leaves begin to grow (Shanker et al. 2005).

### Cadmium (Cd)

Humans, terrestrial creatures, and marine life are all harmed by cadmium (Cd) (Chellaiah 2018). Rises in surrounding Cd concentrations are correlated with increased industrialization and manufacturing density (Nagajyoti et al. 2010). Cd in the atmosphere is caused by mining operations, pesticide sprinkling, and human-induced chemical industries (Rizwan et al. 2016). The accumulation of Cd in the surroundings poses serious health concerns to people, animals, and plants (Abbas et al. 2018b). Cd is easily absorbed by plants from underground water and is then transported by a number of transporters via the phloem to the emerging tip. Transporters, including those found in the rice, OsNRAMP family, OsIRT1, and Arabidopsis AtIRT1 (Songmei et al. 2019; Feng et al. 2021), transfer Cd from soils to the roots. AtHMA4 and AtHMA2 transport Zn and Cd from the root to the shoots, while OsLCT1 transfers Cd from the stems to other tissues (Huang et al. 2015; Hassan et al. 2016). Subsequently, through the phloem, it is transported to various tissues by specialized transporters known as ATPases. Cd destroys plant development physically, phenotypically, and biologically (Abbas et al. 2018b).

Cd reduces plant productivity by lowering biomass, slowing seedling growth, and shortening the tips of roots (Xu et al. 2017). It has been shown that soil toxicity brought on potato crops reduces biomass and crop productivity by Cd (0.06 g/kg) (Nazar et al. 2012). The physiological consequences of Cd exposure include a decrease in chlorophyll concentration, an increase in ROS, and damage to plant membranes and cellular macromolecules. Because of the obstructed mineral absorption in plants, Cd causes chlorosis and necrosis of the leaves. The vital elements of transportation and uptake required for the growth of plants are impeded by Cd. In cauliflower plants subjected to Cd, Jinadasa et al. (2016) found shorter cultivars and indications of leaf insufficiency. Lettuce, radishes, and soybeans have all shown this trait (Wang et al. 2020a). According to the latest research, oxidative damage, ROS generation, and plant growth are all negatively impacted by Cd, posing a threat to world health (Khan et al. 2020). The scientific research supporting the toxic effects of Cd on crops is extensive and includes data on chlorophyll content, growth, germination of seedlings, enzyme function, the consumption of nutrients, ROS, plant transpiration ratios, and crop yield (Zhang et al. 2020).

## Conventional and NPs based techniques for HMMs remediation

### Conventional techniques

#### Physicochemical method

This type of remediation could be carried out with the help of physical and chemical processes like precipitation, ion exchange, filtration, ultrafiltration, reverse osmosis, evaporative recovery, solvent extraction, electrochemical treatment, electrodialysis, electrokinetics, landfilling, chemical oxidation, chemical leaching, chemical reduction, and mechanical separation of metals (Lasat 1999; Asgari Lajayer et al. 2017). Incomplete metal removal, excessive solvent needs, and the production of hazardous waste products are only a few of the drawbacks of such methods. In addition to being harmful to the ecosystem, they also tend to damage the soil. Furthermore, they are time-consuming, costly, and energy-consuming (Gardea-Torresdey et al. 2005).

#### Bioremediation method

Both *in-situ* and *'ex-situ* bioremediation methods exist. Methods of *in-situ* bioremediation remediate contaminants at the site without attempting to remove soil. These methods utilize the metabolic power of the microbial system to eliminate pollutants in the environment, eliminating the need to remove contaminated samples from the environment (Fruchter and Demian 2002). In *ex-situ* bioremediation, soil is removed from its original location, treated, and then replaced (Carberry and Wik 2001). It is more expensive to use *ex-situ* remediation methods than *in-situ* ones.

### NPs based HMMs remediation

Both *ex-situ* and *in-situ* bioremediation methods have advantages and disadvantages. When it comes to bioremediation, phytoremediation is one of the most effective, sustainable, and cost-effective strategies. The term "phytoremediation," derived from the Greek words "phyton" for plants and "remediare" for "to remediate," denotes a process in which certain plants and soil bacteria collaborate to convert poisons into harmless and, often, profitable forms. The concept of employing plants to remove harmful metals from contaminated soils was inspired by the finding of a range of wild plants, many of which are endemic to naturally mineralized soils and accumulate large quantities of metals in their leaves (Baker 1987). The capacity to rapidly absorb chemicals and change them into less harmful metabolites and the ability to endure relatively high levels of xenobiotic chemicals without hazardous consequences are two examples of the plant kingdom's resilience in the face of xenobiotic pollution (Burken and Schnoor 1998).

**Fig. 3:**
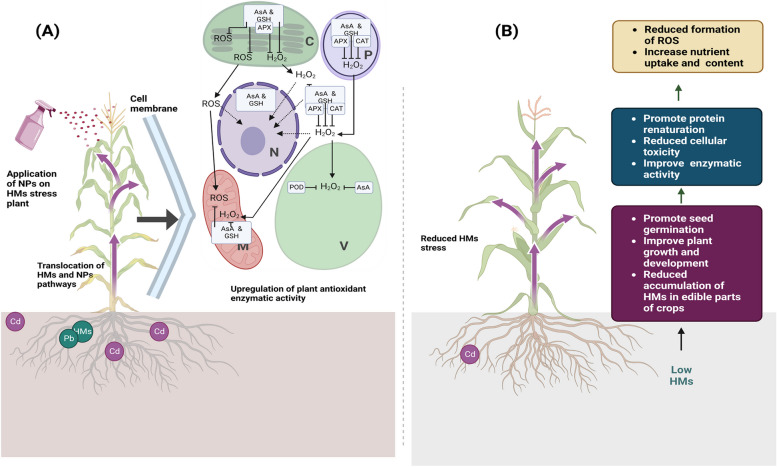
Application of (**A**) NPs and translocation that upregulated the antioxidant defense system that (**B**) mitigated the effect of HMMs on crops

Hyperaccumulator plants, or plants that are hyper-enriched in heavy metals, are those that take up large amounts of heavy metals from their surroundings. This method is becoming increasingly popular for decontamination of the sites tainted with toxic chemicals and metals. Phytoremediation relies on the presence of pollutants in the root zones of plants to be effective. Phytoextraction, phytodegradation, phytostabilization, phytovolatilization, and rhizofiltration are the key processes utilized by plants for remediation. Soil and other environmental HMMs pollutants can be decontaminated by an environmentally friendly and cheap method known as nanoremediation (Baragaño et al. 2020). Through absorption, lowering the hazardous valence to a stable metallic state, and accelerating the process, this unique remediation strategy has been shown to be efficient in the elimination of heavy metals (Mar Gil-Díaz et al. 2014). Adsorption, heterogeneous catalysis, deployment of electrical fields (electronanoremediation), photodegradation, and involvement of microorganisms (nanobioremediation) are just some of the technical processes used in nanoremediation to apply NMs to remove or immobilize HMMs from contaminated soils (Mukhopadhyay et al. 2022a). The removal of HMMs has been effectively applied using a variety of NPs (Fig. 3 and Table 1), including metallic, metallic oxide, carbonaceous, polymeric, and nanocomposites.

**Table 1:** Application of NPs for mitigation of HMMs stress

Plants	Type of NPs	Concentration of NPs	Type of Application mode	Mode of Action	References
*Triticum aestivum*	FeO	100 mg kg^−1^	Soil	Wheat's growth, nutritional content, and antioxidant enzymes were all improved, and the plant's root and shoot Cd absorption reduced by 38% and 72%, respectively	(Manzoor et al. 2021)
*Triticum aestivum*	FeO	100 mg kg^−1^	Soil	The chlorophyll content of wheat's growth was induced and Cd levels were drastically cut	(Adrees et al. 2020)
*Triticum aestivum*	FeO	1–20 ppm	Foliar and soil	Wheat's root, stem, and grain chlorophyll content increased (56%) and its Cd concentration decreased (53%), respectively	(Hussain et al. 2019)
*Oryza sativa*	CeO_2_	200 mg/L	Hydroponic	Growth inhibition, proline levels, and 8-hydroxy-2'-deoxyguanosine levels were reduced	(Wang et al. 2019)
*Triticum aestivum*	ZnO	100 mg kg^−1^	Soil	Reduced Cd accumulation in plants from soil and increased plant biomass and photosynthesis efficiency were all observed in plants subjected to drought stress	(Khan et al. 2019b)
*Oryza sativa*	ZnO	100 mg kg^−1^	Hydroponic	Cd concentration in roots decreased by 61.8%, and Cd concentration in shoots—by 26.3%, whereas As concentration in roots decreased by 39.51%, and As concentration in shoots decreased by 60.2%	(Ma et al. 2020)
*Oryza sativa*	ZnO	5, 10, 15, 20, 25 mg/L	Soil (seed priming)	Root, stem, and leaf HM absorption decreased, leading to greater plant growth. In addition, it resulted in increased plant tolerance and decreased bioaccumulation index and metallothionein (MTs) content	(Akhtar et al. 2021)
*Trigonella foenum-graecum*	ZnO	1, 0.5, 0.25 and 0.10 g/L	Soil	Seed germination, root development, and plant growth were all enhanced by the application of ZnO NPs, outperforming the control and zinc sulfate groups. There was a rise in root tissue Zn content and a decrease in Cr content	(Shaik et al. 2020)
*Leucaena leucocephala*	ZnO	25 mg/L	Hydroponic	It raises the possibility that zinc oxide NPs might be useful in cleaning up media polluted with heavy metals. Additionally, the inclusion of ZnO NPs along with the metals Pb and Cd caused unique changes in the genome	(Venkatachalam et al. 2017)
*Raphanus sativus*	Ag NPs	NA	Pot culture	NPs in wastewater can reduce the uptake of HMMs (Cr, Cu, Fe, Zn, Cd and Pb) by wastewater irrigated food crops	(Zhou et al. 2021d)

The carbon nanotube (CNTs) nano-sponges, for instance, as was reported by Camilli et al. (2014), eliminated the harmful organic solvent dichlorobenzene and the heavy metal Cd from the soil. Generally, iron and sulfur containing nanosponges boost the efficacy of soaking up Cd and other pollutants from soil. Matos et al. (2017) did a similar analysis of CNTs' ability to immobilize HMMs ions (Ni^2+^, Zn^2+^, Pb^2+^, and Cu^2+^) in soil remediation. Based on the findings of this research, even a little addition of CNTs can greatly enhance adsorption capability, leading to more HM immobilization in polluted soils. Soil treatment with Fe_3_(PO_4_)_2_ NPs was shown to be effective in immobilizing Cd by 70% in a separate investigation. This study further revealed that cadmium phosphate synthesis is the key mechanism for decreased bioaccumulation of Cd in soil.

## Nanotechnological approaches for remediation of HMMs

### Methodology for synthesis of NPs

#### Physical methods

The physical approaches used for synthesizing NPs include thermal evaporation, pulsed laser desorption, ball milling, pyrolysis with sprays, electron irradiation, sputter accumulation, lithography techniques, layer-by-layer growth, and the diffusion flame approach (Srivastava et al. 2021). By using these methods, metal NPs are created by the process of evaporation and condensation that takes place at atmospheric pressure in a tube furnace. Deposition, sputtering, ball milling, and plasma-based procedures are some of the physical techniques used to create nanomaterials (Dhand et al. 2015). The majority of these methods produce metal NPs at an excruciatingly slow rate. For example, the production rate of nanomaterials in ball milling methods is 50% (Seetharaman et al. 2018). According to reports, between 6 and 8 percent of the material spewed has a size smaller than 100 nm, creating a broad particle size distribution. Plasma techniques and laser ablation need a lot of energy. Due to their higher consumption of energy, size dispersion, and sluggish manufacturing rate, the majority of physical techniques are unaffordable (Seetharaman et al. 2018).

#### Chemical mthods

Among the chemical processes suggested for creating NPs are pyrolysis, polyol synthesis, sol–gel technique, hydrothermal synthesis, microemulsion, chemical reduction, and chemical vapour deposition (Darroudi et al. 2013). Furthermore, the use of hazardous materials and chemicals during the manufacturing stage produces waste that is dangerous for both humans and the environment (Darroudi et al. 2013; Barzegar et al. 2023). Consequently, these NPs can only be used in the biological domain.

#### Biological or green methods

Green nanotechnology is quickly evolving as a technique for creating innovative NPs with low ecological impact. NPs can be synthesized by biological processes using different natural stabilizing and reducing agents. This is a less expensive and more environmentally friendly option that uses chemical and physical processes and requires very little energy or dangerous materials. Single-celled organisms such as bacteria and fungi are used in the bottom-up biological production of NPs, as are multicellular species like fungi, algae, or plant components (Mittal et al. 2013; Panpatte et al. 2016; Singh et al. 2016; Patil and Chandrasekaran 2020a).

1. Bacteria

Industrial uses of biotechnology that heavily rely on specific strains of bacteria include genetic engineering, bioremediation, and bioleaching (Hoffmann et al. 1995). Because they are endowed with the ability to reduce metal ions, bacteria are important options for manufacturing NPs (Birla et al. 2009; Agarwal et al. 2017). Several forms of bacteria are employed in the synthesis of metals and NPs. It has been widely studied how prokaryotic bacteria and actinomycetes may be used to synthesize metal and metal oxide NPs. Yet, during the production of Cd NPs, glutathione and cysteine desulfhydrase in *E. coli* have been largely responsible for the creation of spherical shapes (Eroglu and Metin 2023; Wang et al. 2023a). The ultimate size of the NPs may depend on how they interact with biologically active substances and whether they are located extracellularly or intracellularly (Thakkar et al. 2010). Larger NPs are usually formed by external synthesis in bacteria as opposed to intracellular synthesis. In relation to selenium, lead sulfide, zinc sulfide, and ferrous oxide, bacterial systems have been employed in the production of nanomaterials (Patil and Chandrasekaran 2020).

It has been demonstrated that a wide variety of ecologically active chemicals may stabilize and lower NPs. The proteins presented in the cytoplasm and cell wall of bacteria, such as tryptophan and tyrosine containing proteins, can stabilize and decrease bacterial NPs. Furthermore, certain sugars have stabilizing and lowering qualities, such as aldose and ketose. The amino acids found in cells and inside their walls function as a barrier to lessen the likelihood that NPs will be harmful to mammalian cells (Markus et al. 2016). These bioactive compounds, which are present in various types of bacteria, have the ability to interact with metal ions and reduce their size. As a result, the metal ions interact with one another to create more intricate structures, such as spherically shaped NPs. Because microorganisms are easy to control, the production of bacterial NPs has gained great popularity (Adelere and Lateef 2016). Several types of bacteria are used in the formation of different NPs, including *Aeromonas sp. SH10, Arthrobacter gangotriensis, Bacillus amyloliquefaciens, Bacillus cereus, Bacillus cecembensis, Bacillus indicus, Corynebacterium sp. SH09, Enterobacter cloacae, Escherichia coli, Geobacter spp., Phaeocystis antarctica, Pseudomonas proteolytica, Lactobacillus casei, and Shewanella oneidensis* (Singh et al. 2018).

2. Fungi

The manufacture of metal- or metal oxide-based NPs by fungi is an important method for producing monodisperse NPs with distinct morphological characteristics. They are better biological agents for the synthesis of metal and metal oxide NPs because they have several intracellular enzymes (Hoffmann et al. 1995). Competent fungi are able to synthesize significantly higher amounts of NPs than bacteria (Hoffmann et al. 1995). Due to the presence of different proteins, reducing components, and enzymes on the outermost layer of their cells, fungi have numerous important uses (Narayanan and Sakthivel 2011). Enzyme reductase, which induces enzymatic reduction and is present in the fungal cell wall and cells, is crucial to the biological process of metallic NP synthesis (Mohanpuria et al. 2008). Various types of fungi are used in the synthesis of gold, silver, zinc oxide, and titanium dioxide NPs.

Furthermore, it is being shown that proteins contained within cells can contribute to the synthesis of NPs. It has been reported that the intracellular enzymes found in *Verticillium luteoalbum* and *Trichothecium* produce gold nanorods and nanospheres (Gericke and Pinches 2006; Ahmad et al. 2006). Fungi-generated NPs offer a wide range of potential applications, from optoelectronics to medicine, similar to those created by other ecologically benign technologies (Birla et al. 2009). More study on NPs is necessary because of their fascinating capabilities in both scientific and therapeutic domains. It has been observed that NPs made from consumable mushroom extracts have chemotherapeutic characteristics that are comparable to those of the extracts themselves (Philip 2009). The residues of amino acids found in fungi, such as cysteine, may contribute to the production of NPs (Philip 2009). Similar to bacteria, fungi employ the amino acids found in their cell walls for capping and stabilizing the cell walls. Additionally, these NPs are harmless when used for therapeutic purposes, in contrast to those that are synthesized biologically (Philip 2009).

3. Yeast

Yeasts are eukaryotic, unicellular microorganisms. There are now over fifteen hundred known varieties of yeast (Yurkov et al. 2011). Using yeast, multiple labs have demonstrated the effective manufacturing of NPs. It has been demonstrated that *Saccharomyces cerevisiae* broth and silver-tolerant yeast may biosynthesize Ag and Ag NPs (Patil and Chandrasekaran 2020a). Massive amounts of metal NPs are produced by a wide variety of organisms. Yeast cells allow for the synthesis of a wide range of nanosystems which would be hard to do with just bacteria. The production of gold, silver, ferrous oxide, cadmium sulfide, lead sulfide, antimony, and selenium NPs is done using yeast species. The production of NPs can be carried out with living cells or with cell extracts, including typical nanomaterial composites like silver and gold. Using proteins obtained from industrial yeast, silver chloride NPs were effectively generated (Abdel-Hadi et al. 2014). In order to create the NPs, commercially available yeast extracts and precursor solutions were first incubated for 24 h (Abdel-Hadi et al. 2014). Following incubation, the NPs-containing mixture was collected and thoroughly filtered to get rid of any impurities. Additionally, they showed that primary amines of certain proteins buried in the yeast cell wall are what trigger silver chloride reduction into NPs. Additionally, it was shown that these NPs had anti-mycobacterial properties (Huston et al. 2021). NPs might be produced by MKY-3 yeast cells that are resistant to silver (Philip 2009). These nanomaterials' sizes and shapes vary depending on factors that govern their synthesis, such as pH and silver chloride concentration.

The research findings indicate that secreted biochemical reducing agents were responsible for the extracellular reduction of silver chloride (Philip 2009). Although there are several methods for yeast to generate NPs, extracellular production is not qualified as one of them. A large number of the study groups said that the construction of the nanosystem was carried out by enzymes that were already present in the cell, indicating that their models of synthesis occurred within cells. According to their earlier studies, *Schizosaccharomyces* pombe and *Torulopsis* sp. were able to produce lead sulfide and cadmium sulfide NPs intracellularly, respectively (Kowshik et al. 2002). As compared to their previous studies, Kowshik et al. (2002) showed that intracellular manufacturing of NPs might be caused by a particular phytochelatin synthase species. Remarkably, manufactured NPs functioned wonderfully across a range of biological situations, according to nearly all of the examined publications (Kowshik et al. 2002). The phrase "biological applications" encompasses a broad variety of potential uses due to the adaptability of NPs. *Saccharomyces cerevisiae* produces silver NPs that eliminate mycobacteria in culture (Sivaraj et al. 2020). Yeast can produce proteins that include the required amino acids, similar to other living organisms, which helps to diminish and stabilize the NPs. Quinones, a class of organic substances formed from aromatic substances, are quite different from yeast and have been scientifically demonstrated to help in the formation of NPs. The activation of oxidoreductases allows them to start lowering the metal ions as the pH level within the cell becomes more basic. Quinones and potent nucleophiles with redox characteristics are helpful in catalyzing the conversion of disordered metal ions into complicated NPs (Jha et al. 2009; Faramarzi et al. 2020).

4. Algae

Although they are not classified as plants, algae are a class of eukaryotic organisms that can carry out photosynthesis. These single and multicellular algae species do not have stems, leaves, or vascular structures, which are features of plants, yet they are nevertheless chlorophyll generators that flourish in watery conditions. Certain types of algae, like spirulina, can be utilized medically because of their greater amounts of natural vitamins and minerals, whereas others, like anabaena, are deadly if consumed because of their cells and poisons (Carmichael et al. 1990; Khan et al. 2005). In recent times, it has been revealed that a wide variety of algae may catalyze the creation of nanomaterials (Khan et al. 2005). Various species of algae are crushed into a small powder, appropriately dried, and subsequently combined with water to create nanomaterials. After letting them to rest for around a full day, the filtration procedure starts. After the ingredient has been filtered, it is combined with the nanomaterial's precursor and incubated at a certain temperature without being interrupted. A shift in color takes place, indicating the creation of nanomaterials. According to Senapati et al. (2012), a small number of algae species, such as *Tetraselmis kochinensis*, are known to promote the production of intracellular gold NPs via enzymes found across the cell wall and within the cytoplasm. On the other hand, a comparison of two distinct categories of algae reveals a multitude of choices about the NPs. However, the comparison of two algal species groups shows a wide range of options with context to the NPs morphology.

According to Mukherjee et al. (2021), species such as *Cystophora moniliformis*, *Leptolyngbya valderianum,* and *Scenedesmus sp*. catalyze the synthesis of NPs, resulting in NPs with a spherical shape. According to Sinha et al. (2015), *Pithophora oedogonia* possesses the ability to produce both hexagonal and cubic-shaped AgNPs,along with nanospheres (Sinha et al. 2015). Algae and green agents possess more than just an appearance, as their bioactive substances are extensively similar (Mukherjee et al. 2021). Several kinds of algae produce NPs, and a large portion of these NPs are stabilized and reduced by proteins found in the cytoplasm or on the cell membrane, as well as by enzymes (Mukherjee et al. 2021). Finally, the majority of NPs produced by algae are efficient bactericides. When it comes to the synthesis of NPs, algae and bioactive compounds from other groups are a bit comparable but also somewhat distinct. Since the walls of algae include polysaccharides and protein debris, they have the capacity to reduce and stabilize NPs. One essential characteristic of algae that may be greatly exploited is their phytochemical variety. A wide variety of bioactive substances, such as phenolic molecules, alkaloids, amino acids, carbohydrates, sterols, flavonoids, tannins, and saponins, are present in certain algae, such as *Sargassum tenerrimum*. Following the process of purification, these chemicals have a distinct role in modifying the size, shape, and function of nanomaterials. Algal characterization is an initial move towards pursuing future research and potential use in nanomaterial synthesis (Saleh 2020).

5. Plants

Plants may retain various quantities of heavy metals within their tissues. Since they are a considerably more effective, simple, affordable, and feasible option to conventional preparation techniques for the formation of NPs, biosynthetic approaches utilizing plant extracts have drawn greater attention (Akhtar et al. 2013). According to Singh et al. (2018), plants can be employed in a "one-pot" synthesis method to concurrently decrease and stabilize metallic NPs. Scientists have produced metal/metal oxide NPs and explored their potential applications using extracts of plant leaves in an ecological manufacturing procedure (Singh et al. 2018).

There have previously been reports of NPs of various metals, including copper, gold, and selenium. It has been discovered that a number of plant-based compounds include reducing, capping, and stabilizing chemicals that may be used to create Ag NPs (Singh et al. 2018; Huston et al. 2021). The examples of substances that may function as stabilizing, capping, and reducing agents include vegetable oil, tea polyphenols, *Carpesium cernuum*, *Cannabis sativa*, and black currant (Huston et al. 2021). The production of colloidal Ag NPs from tea leaves high in polyphenols was reported by Moulton et al. in 2010 (Moulton et al. 2010). Their synthesis approach has similarities to other approaches that have been earlier documented. The group of people was offered tea powder, which was created by grinding dried tea leaves. They were then steamed and drained, and subsequently, tea powder was mixed with silver nitrate to create Ag NPs, which were later confirmed by Transmission Electron Microscopy (TEM). Subsequently, tests for the viability of cells and the integrity of the membrane were employed to evaluate the toxic effect of NPs on living things. According to Moulton et al. (2010), the NPs were discovered to be harmless and perhaps biologically compatible, which was a promising discovery (Moulton et al. 2010). A different plant component that is common in many houses is vegetable oil. For a minor variation in the metallic NPs green synthesis, Kumar et al. (2008) used free radicals present in home paints derived from specific vegetable oils (Kumar et al. 2008). In order to create Ag NPs, the researchers employed silver benzoate, a well-known silver salt, together with the conversion of free radicals during the oxidative oil drying procedure.

The researchers also employed alkyd resins as protecting reagents and the aldehydes and fatty acids derived from the oils as reaction-stabilizing agents. As a result of their response, antibacterial paints incorporating Ag NPs were created (Kumar et al. 2008). Another plant component that may be used in the process of creating NPs is aloe vera. Sunburns are now frequently treated using aloe vera, which has been traditionally employed for millennia in complementary therapies to treat a variety of ailments. Se NPs with antifungal and antimicrobial properties were effectively made using aloe vera (Fardsadegh and Jafarizadeh-Malmiri 2019). Although the hydrothermal procedure is praised as a traditional synthesis method, it is really far more environmentally friendly (Fardsadegh and Jafarizadeh-Malmiri 2019). Similar techniques that are used to harness the bioactivity of plant leaves may also be applied to spices. Indonesian natives produce the aromatic fruits of the *Myristica fragrans* tree. The fruit generates the spices mace and nutmeg after it gets dried and processed. Metallic NPs can be produced from the pericarp, the part of the fruit that is not a seed. After being dried and crushed, they were put into water and brought to a boil using either cupric oxide or silver nitrate. Subsequent investigation revealed that phenols, quercetin, and flavonoids from *Myristic fragrans* were essential for the stabilization and reduction of the NPs (Sasidharan et al. 2020). Furthermore, it is well recognized that Ag NPs are extremely effective in breaking down bacterial cell walls. Furthermore, Cu NPs worked effectively with catalysts during the production of triazole rings (Sasidharan et al. 2020).

### Uptake and translocation processes of NPs

#### Interaction of NPs with plants

For NPs to enter the symplastic pathway, plant cells must internalize them and pass across their plasma membrane. A number of pathways support this mechanism, but our knowledge of them is limited to animal cells (Avellan et al. 2017; Hong et al. 2021). Endocytosis, which includes the invasion of the plasma membrane and the formation of vesicles that can migrate to specific cell areas, is a potential way that NPs could become anchored within cells (Wang et al. 2013b; Bharali et al. 2017; Usman et al. 2020).

There is an additional method wherein certain nanomaterials cause the plasma membrane to rupture, creating holes that allow for direct access inside the cell without the need for encapsulation in organelles (Zhao et al. 2012b). Additionally, NPs have the ability to attach to nearby proteins, such as those found in cell membranes, which serve as effective carriers for NP absorption and internalization inside the cell (Kapilan et al. 2018). For example, aquaporins have been proposed to function as NP carriers (Maurel et al. 2015). Yet, unless changes are introduced to improve the pore dimension, it is doubtful that they will operate as passageways for NP entry because of their modest size of pore (varying between 2.8 and 3.4 Å) (Sohail et al. 2022). Plant cells may also receive NPs through plasmodesmata, which are specialized structures for intercellular movement; however, plasmodesmata need NPs that are already existing inside the symplast. This process is especially important for the transfer of NPs via the phloem (Sun et al. 2013). While ion channels have previously been suggested as potential NP entrance points, it is extremely unlikely that NPs could pass them efficiently without significant changes due to their normal size of approximately 1 nm (Paramo et al. 2020; Wang et al. 2013c; Pérez-de-Luque 2017; Hu et al. 2020). It is important to highlight that research on the molecular mechanisms involved in NP absorption in plant cells is still ongoing, and more research is necessary to fully understand these biological procedures.

#### Uptake and translocation of NPs from the soil

The soil's pores and root systems are the primary and the most significant entrance sites for NPs into the soil (Khan et al. 2019a). The best places for NP uptake within the rhizosphere are the lateral root system and developing root hairs (Khan et al. 2019a). The first point of contact between NPs and plant roots occurs when the NPs are adsorbed onto the outermost layer of the roots by their root systems. Because root hair openings secrete phytochemicals that cause the root surface to become negatively charged, positively charged NPs are more inclined to accumulate and settle rapidly. (Lv et al. 2019; Wang et al. 2023b). Once NPs reach plant tissues, they may travel easily across them, utilizing apoplastic and symplastic movements, passing through organic acids and mucus, among other compounds (Flowers 2004; Zhao et al. 2012a; Petosa et al. 2017; Gong et al. 2020). According to Sattelmacher (2001), the radial movement of NPs within vascular tissues is facilitated via the apoplastic route. Water and additional substances are exchanged between the neighboring cell's cytoplasm and symplast (Wang et al. 2013a). It is possible that the emergence of lateral roots will produce a fresh adsorption surface through which NPs can enter the root stream (Zhang et al. 2015; Peng et al. 2016).

Both physically and functionally, the root epidermis mimics the leaf surface. However, the outer layers of the root hair and root tip epidermis of both primary and secondary plant roots are still developing. Here, the exposure of NPs causes them to immediately enter and come into contact with the root epidermis (Rajput et al. 2018; Singh et al. 2022). Root epidermal cells have a permeable cell membrane that allows certain nutrients as well as water to pass through. Tightly compacted cell walls in root cells serve as a barrier against larger and unwanted particles (Pérez-de-Luque 2017). The physical obstacles that the plasma membrane of the root cell forms between the roots and the soil are impermeable to hydrophilic and macromolecules, but small molecules such as steroids can pass past them. The GLUT1-4 transporter/carrier and channels like ATPase are used to transfer such molecules throughout the cell membrane (Zulfiqar et al. 2019a; Zulfiqar and Ashraf 2021). Those membrane channels and transporters could also be involved in the movement of NPs from the soil towards the root cells (Ma et al. 2023; Wang et al. 2023b). When a root does not possess an exodermis, NPs may penetrate the xylem, or prominently positioned vascular column (Péret et al. 2009). Further investigations have demonstrated that certain NPs may disrupt the plasma membrane and induce new holes in the epidermal cell wall, which allow bigger NPs to pass through easily and effectively (Lv et al. 2019). Upon introduction of NPs to plant cells, there are many pathways by which the cells can absorb them (Zhang et al. 2015; Peng et al. 2016). Some of the key processes are physical injury, protein attachment to cell membranes, endocytosis, and the ion route.

According to Servin et al. (2013) and Slomberg and Schoenfisch (2012), accumulated NPs in plants are different from injected NPs (Slomberg and Schoenfisch 2012; Servin et al. 2013). NPs such as TiO_2_ and SiO_2_ are found in plants in their natural state because of their morphological and biological stability, but ZnO, NiO, CuO, Yb_2_O_3_, CeO_2_, and La_2_O_3_ are transformed. For example, Zn has been absorbed and transported as Zn^2+^ in maize after treatment because ZnO NPs change in the rhizosphere (Lv et al. 2019). All of the zinc found in maize aerial shoots and subterranean roots, however, was found mostly as ZnPO_4_. According to Dimkpa et al., wheat fields injected with ZnO NPs and CuO NPs collected ZnPO_4_ and Cu(I)-sulfur complexes (Dimkpa et al. 2019). Cu (II) reduction to Cu (I) was evident in the rice and maize crops. Cu reduction (II) is the outcome of CuO NPs moving from rice root zones to shoots. Cu (II) has the tendency to bind with citrate and cysteine, along with being reduced to Cu_2_O (Zhang et al. 2015; Peng et al. 2016). It was found out that the rhizosphere and various intercellular plant areas contain the converted final products of CeO_2_, Yb_2_O_3_, and La_2_O_3_ NPs (Zhang et al. 2012). NPs were applied to a hydroponic cucumber culture, and it was discovered that 7 nm CeO_2_ NPs were converted to Ce (III) and accumulated as CePO_4_ in the intercellular cavities of the roots. The natural acid-rich root exudates facilitated CeO_2_ NP dissolution. The change in locations of CeO_2_ NPs within cucumber plants was subsequently validated by Ma et al. (Rossi et al. 2017). The detection of a buildup of Ce (IV) and Ce (III) in response to root exposures to NPs clarified the role of the rhizosphere in the breakdown of CeO_2_ NPs, although this was not the case in the instance of foliar application. Studies revealed that in cucumbers, cedar travels as Ce (IV) and Ce (III) through the xylem from the roots to the shoots, but it travels as CeO_2_ from the shoots to the roots.

### Foliar application of NP uptake and translocation

NPs are applied topically onto the leaf surface, where they settle and get taken up by the plants through specific attachments such as stomatal holes, hydathodes, or trichomes. The waxy layer of cuticle that covers the leaf epidermis is mostly made up of wax, cutin, and pectin. It functions as an essential natural barrier that keeps NPs out while keeping growing leaves from losing water (Peng et al. 2015). On the other hand, the waxy stratum corneum possesses two unique channels: one is hydrophilic while the other is lipophilic. According to Eckert and Goldbach (2008), the hydrophilic and lipophilic channels have widths between 0.6 and 4.8 nm. (Eichert and Goldbach 2008). Through the hydrophilic channels, hydrophilic NPs with a geometrical dimension of less than 4.8 nm can diffuse (Banerjee et al. 2019). Lipophilic NPs are taken up by foliage by penetration and diffusion via the lipophilic channels within the cuticle (Bussières 2014). Using high-resolution confocal fluorescence microscopy, P. Hu et al. (2020) have demonstrated that carbon dots as tiny as 2 nm may penetrate the cuticular route and reach cotton leaves. But the modest size of the pore channels in the cuticle limits the absorption of NPs via the epidermis.

NPs have a tendency to gather in the epidermis and veins of leaves after being sprayed on them. Meanwhile, it has been demonstrated by multiple investigations that NPs are transposable to different plant tissues. The study team hypothesized that plants may uptake NPs via the stomatal channel (Wang et al. 2013d). NPs are sprayed onto the outermost layer of leaves, where they are taken up by plants through stomata, trichomes, or hydathodes. The primary components of the waxy cuticle found on leaf epidermis are wax, cutin, and pectin. According to C. Peng et al. (2015), it acts as a crucial organic obstacle to keep water from evaporating and NPs from penetrating (Peng et al. 2015). The waxy stratum corneum is divided into two distinct channels: lipophilic and hydrophilic. The diameter of hydrophilic or lipophilic channels can vary from 0.6 to 4.8 nm (Eichert and Goldbach 2008). The hydrophilic channels allow hydrophilic NPs less than 4.8 nm to diffuse (Banerjee et al. 2019). Lipophilic NPs can enter and diffuse into leaves more easily because of the lipophilic pathways found in the cuticle (Bussières 2014). Nonetheless, the pore channels' tiny apertures function as a cuticle regulator, limiting the best possible intake of NPs via the epidermis. Investigations reveal that when NPs are introduced to a leaf's surface, they build up in the outermost layer of the epidermal and vascular tissues of the leaf. In the meantime, a great deal of research has shown that NPs are translatable to different plant tissues. The researchers in the investigation hypothesized that plants could take up NPs via their stomata.

The plant species is one of the many elements that affect the absorption, movement, and accumulation of foliar NPs. The distinct placement of stomatal holes in each might explain the variation in absorption behavior between dicotyledons and monocotyledons. Higher leaf surface area, smaller petioles, and a smaller number of veins all contribute to NP accumulation in plants. The lifespan and developmental stage of the NPs determine how much foliar supplemental nutrition is applied to the leaves. Foliar NP absorption was inhibited by physiochemical characteristics, including development stage, epidermal organization, NP size, and leaf cross-sectional surface area (Ullah et al. 2020). While hydrophilic nanosuspensions are readily taken via cell walls, lipophilic epidermal wax facilitates the absorption of hydrophobic NPs. Furthermore, the organic and inorganic compounds produced by phyllosphere microorganisms may acidify, reduce, and chelate NPs in the meantime, influencing NP entrance within leaves. For example, humic acid has been linked to the chelation of ZnO NPs (Rahale et al. 2021), whereas Fe NPs are chelated by ethylene diamine-N and N-bis(2-hydroxyphenylacetic acid) (Jalali et al. 2016). Because metal- and carbon-derived NPs attach to the thiol group of different chemical molecules located in vacuoles after reaching the leaves, they have a reduced capacity to be effectively absorbed and transported in plants. As a last line of protection against NP translocation, the endodermal casparian strip keeps it from getting into the circulation (Xie and Yang 2020).

Several abiotic environmental factors, such as humidity, light, and temperature, might have a negative impact on NP absorption when applied topically. For example, extreme temperatures may cause a leaf's epidermis to shrink, while reduced moisture and shadow may induce stomata to shut. The osmotic capacity of the leaf is lowered by elevated humidity levels, which promotes NP absorption. Abiotic variables affect NP absorption by foliar application and impede the growth of the epidermis (Fatemi et al. 2020). Whenever the epidermis becomes thinner and less strong in response to certain abiotic stresses (shade, high temperature, and low humidity), the stomatal mechanism shuts down and the aerial absorption of NPs decreases. A high humidity level encourages the absorption of NPs directly as a result of their effect on the leaf surface's osmotic capacity (Shahid et al. 2017). Abiotic variables like sunlight and temperature have an impact on NPs' foliar absorption, which subsequently in turn has an impact on the efficiency of photosynthesis and the growth of the leaf epidermis (Fan et al. 2020). According to Alshaal and El-Ramady, rainfall decreases the sprinkled NPs' uptake by washing them away (Alshaal and El-Ramady 2017).

### Mode of action of NPs for mitigation of HMMs: insights into mode of action

The processes through which plants manage HMMs and NPs depend critically on interactions with the environment, plant physiology, and the stability of HMMs and NPs. An NP's behavior is affected by its properties (Sabo-Attwood et al. 2012). The morphological and physiochemical characteristics of NPs also play a role in their uptake. Reducing the bioavailable HMMs concentration in the soil, strengthening the plant's defense mechanisms, improving physiological functions, and regulating the expression of HMMs transport genes are all strategies that can help plants cope with HMMs stress (Cao et al. 2020; Rincón-Torres et al. 2021). Plant cells allow NPs access through both channels in the plasma membrane and direct cell membrane penetration. These NPs play a communication role, stimulating the expression of genes involved in the plant's response to stress.

Additionally, NPs can alter or absorb HMMs in the soil, decreasing their bioavailability and mobility. For instance, one study found that cadmium mobility decreased when Fe_3_O_4_ NPs were used (Sebastian et al. 2019). Manzoor et al. 2021 reported that the strong surface reactivity, electrostatic attraction, large surface area, and presence of capping molecules in FeO NPs allowed them to absorb Cd from the soil efficiently. Metal ion concentrations in plants are lowered because Cd^2+^ and FeO NPs fight for the same transporter pathway in cells (Manzoor et al. 2021). In addition, Noman et al. (2020) conducted an analysis and found that the electrostatic attraction, reactivity, and large surface area of Cu NPs impeded Cd translocation from the soil to the wheat plants' aerial portions. The capping molecules on biological Cu NPs may have helped in the immobilization of Cd in the soil. The proteins that coat Cu NPs may have aided in Cd immobilization. The increased demand for Cu-bound nutrients and a subsequent growth spurt in wheat can be attributed to this conflict.

In order to facilitate the transformation of cadmium into more stable forms, research has shown that mercapto Si NPs can play a crucial role (Wang et al. 2020b). We shall go into more depth on how HM toxicity causes an increase in intracellular ROS later. In the context of HMMs, the elements that generate ROS are unique. An indication of HM toxicity is an increase in intracellular ROS generation (Cho and Seo 2005; Skórzyńska-Polit et al. 2006). However, Cd, a redox-inactive HMMs, may only directly produce ROS by inactivating enzymes and increasing the production of LOX in plant tissues, whereas Cu can do so directly. Plants' antioxidant machinery is triggered in response to HMMs stress. However, ROS can disturb cellular activities, which, when combined with ethylene and jasmonic acid (JA), can promote senescence (Gamalero et al. 2009). When ROS build up inside a cell, they are scavenged by antioxidant enzymes such as peroxidase (POD), superoxide dismutase (SOD), ascorbate peroxidase (APX), catalase (CAT), glutathione peroxidase (GPX), and glutathione reductase (GR) (Fig. 3).

Low-molecular-weight non-enzymatic metabolites can successfully neutralize the ROS in addition to enzymes (Ullah et al. 2020). The body's ROS clearance mechanisms are triggered in the face of adversity. To combat the effects of oxidative stress, plants synthesize shikmate-phenylpropanoid and alanine and metabolize galactose, ascorbate, and aspartic acid (Hussain et al. 2016). CeO_2_ NPs, Mn_3_O_4_ NPs, and Fe_3_O_4_ NPs, which are activated by antioxidant enzymes, have been found to reduce crop losses (Konate et al. 2017).

Using a foliar application of CeO_2_ NPs (200 mg/L), researchers were able to successfully stimulate the antioxidant defense system and prevent Cd accumulation in rice grown under hydroponic conditions (Wang et al. 2019). Ji et al. (2017) found that TiO_2_ NPs had a negligible impact on rice seedling biomass when grown in hydroponic conditions. The addition of TiO_2_ NPs mitigated the negative effects of Cd stress on rice seedlings, as evidenced by improvements in root length, plant height, hormone level, antioxidant enzyme activity, and other physiological parameters.

Plants respond to HMMs stress by producing amino acids, proteins, genes, and signaling molecules that are specific to the stress (Dalcorso et al. 2010). Recent research has also shown that exposure to HMMs increases the production of heat shock proteins (HSPs) (DalCorso et al. 2008; Gupta 2010; Solórzano et al. 2020).

There is evidence that proline (Pro) plays a role in HMMs detoxification, since a higher Pro level was detected in the Cd hyperaccumulator plant *Solanum nigrum* compared to the nonaccumulator plant (*Solanum melongena*) (Sun et al. 2007; Zhao et al. 2011). Chelating substances such as phytosiderophores, nicotianamine, and organic acids are secreted by roots and may affect HM uptake (Dalcorso et al. 2010). Mitogen-activated protein kinase (MAPK) cascade signalling pathways show up in different stress conditions like salinity and drought; similarly, it is also observed to respond to HMMs stress (Dalcorso et al. 2010; Singh et al. 2023b). When seedlings of the lucerne (*Medicago sativa* L.) plant are exposed to Cu or Cd ions, this leads to the activation of four different MAPKs, e.g., SIMK, MMK2, MMK3, and SAMK. SAMK, MMK2, and MMK3 were activated more slowly by Cd stress, while SIMK, MMK2, and MMK3 were swiftly activated by Cu stress (Jonak et al. 2004; Gupta 2010). Recent reports also suggested that the application of NPs can also regulate the expression of these genes that are related to HMMs stress (Morales-Díaz et al. 2017; Manzoor et al. 2021; Farajzadeh Memari-Tabrizi et al. 2021; Zulfiqar and Ashraf 2022). Researchers found that nanoscale zero-valent iron (nZVI) increased SOD and POD in sunflower leaf tissue, decreased HM formation, and stimulated plant development (Michálková et al. 2017). The gene expressions of Fe transporters (IRT1, IRT2, YSL2, YSL15) responsible for both Fe and Cd absorption were shown to be dramatically down-regulated after nZVI (100 mg/L) was applied to rice seedlings (Guha et al. 2020). Cd was sequestered in vacuoles due to the overexpression of the OsVIT1 and OsCAX4 genes. The addition of Si NPs significantly enhanced the percentage of viable rice cells in Cd solution (Cui et al. 2017). Possible explanations of this behaviour of rice cells include up-regulation of Si uptake (OsLsi1) genes and the down-regulation of Cd uptake and transport (OsLCT1 and OsNramp5) genes (Cui et al. 2017).

## Conclusion

The present review indicates rapid increaese of metal and metalloid pollutants in environmentespecially soil system due to releases of various natural and anthroponigenic sources. This is an alarming issue for food safety and human health. These elements also hamper soil health which resulted in low quality food produces. The convenational and ongoing discussed approaches for elimination or stabizing these elements in soil and preventing their accumulation in plant tissues were discussed in detail, revealing that these technologies have limitations and are not as effective. However, discussed nanotechnological approaches proved to be effective and ecofriendly with promising outcome to eliminate metal and metalloid pollutants. In summary, nanoparticle-based remediation shows great promise for addressing environmental contamination. The review highlights key mechanisms, recent advancements, and the importance of considering environmental impacts. While acknowledging the potential, a balanced approach is essential, considering both efficacy and environmental concerns. Future research should prioritize scalable and cost-effective technologies, fostering interdisciplinary collaboration for practical application. This review paves the way for sustainable and responsible use of nanoparticles in metal and metalloid pollutants remediation.

## Data Availability

Not applicable.

## Abbreviations

HMMs: Heavy metals
SOD: Superoxide dismutase
POD: Peroxidase
CAT: Catalase
ROS: Reactive oxygen species
NPs: Nanoparticles
GPX: Glutathione peroxidase
